# A case report and literature review of cutaneous intravascular large B-cell lymphoma presenting clinically as panniculitis: a difficult diagnosis, but a good prognosis^☆^^☆☆^

**DOI:** 10.1016/j.abd.2020.08.004

**Published:** 2020-11-18

**Authors:** Deniz Bayçelebi, Levent Yıldız, Nilgün Şentürk

**Affiliations:** a Department of Pathology, Faculty of Medicine, Ondokuz Mayıs University, Samsun, Turkey; b Department of Dermatology, Faculty of Medicine, Ondokuz Mayıs University, Samsun, Turkey

**Keywords:** Intravascular large B-cell lymphoma, Scleroderma, localized, Panniculitis, Skin neoplasms

## Abstract

Intravascular large B-cell lymphoma is a rare, non-mass-forming, extranodal large B-cell lymphoma subtype characterized by the presence of tumor cells in the lumens of vessels. It is divided into two major types: classical and Asian. Patients presenting only with skin involvement are mostly female, at a younger age than classical intravascular large B-cell lymphoma patients, and have a better prognosis. Since the diagnosis of cases with isolated skin involvement is difficult, keeping this entity in mind, performing a careful microscopic examination, and applying new, effective treatment regimens will make it possible to achieve better clinical outcomes in these cases.

## Introduction

Intravascular large B-cell lymphoma (IVBCL) is a rare, non-mass-forming, extranodal large B-cell lymphoma subtype.^1^ It is characterized by tumor cells accumulation in the lumens of small and medium vessels.^1^ This tumor was included in mature B-cell neoplasms in the 2008 World Health Organization (WHO) classification.^2^ In this study, IVBCL will be reviewed in light of the literature on a case presenting with unusual clinical features.

## Case report

**Figure 1:**
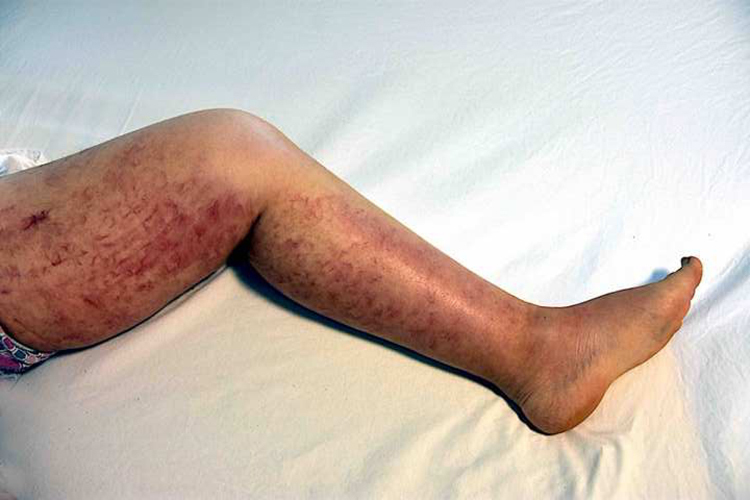
Macroscopic appearance of lesions on legs.

**Figure 2:**
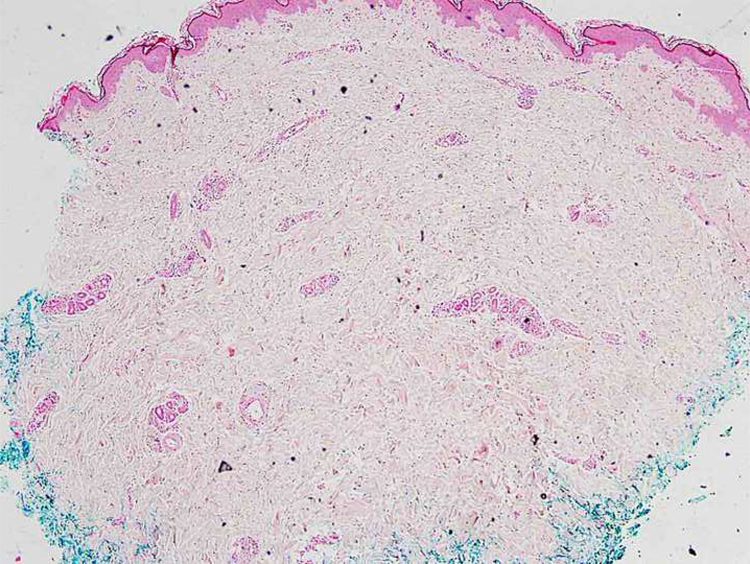
Histopathological examination at low magnification, which was initially interpreted as morphea (Hematoxylin & eosin, ×100).

**Figure 3:**
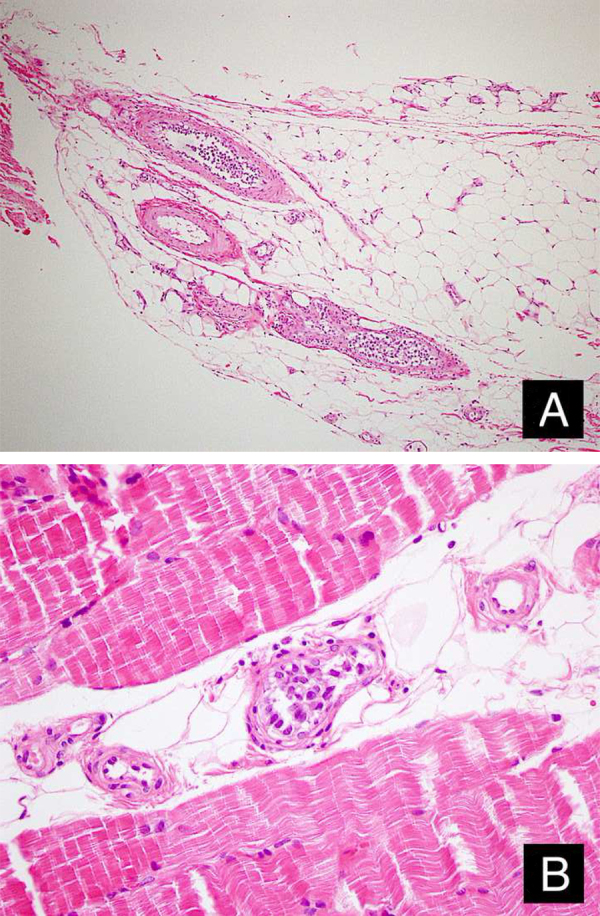
Tumor cells in the lumens of vessel in the subcutaneous adipous tissue (A: Hematoxylin & eosin, ×100) and muscular tissue (B: Hematoxylin & eosin, ×400).

**Figure 4:**
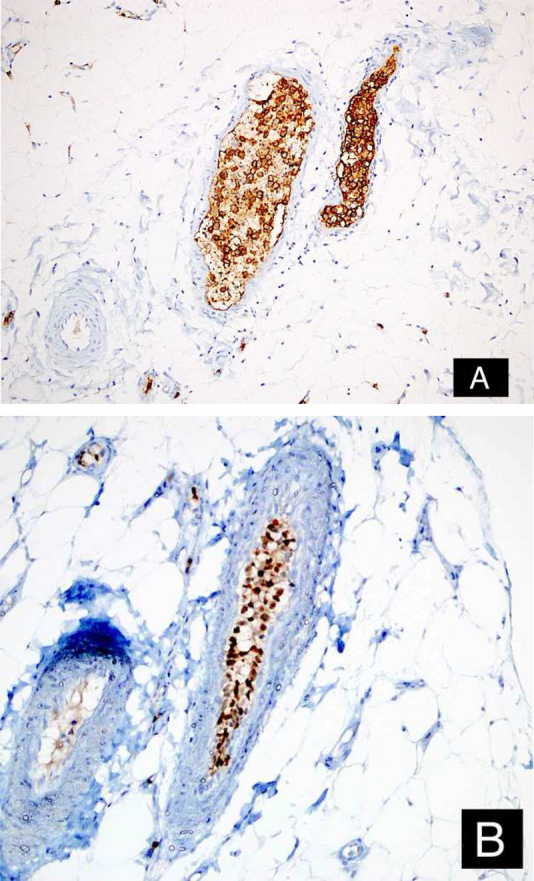
Tumor cells positive with CD20 (A: DAB, ×200), and MUM-1 (B: DAB, ×200).

A 46-year-old female patient presented to the dermatology polyclinic with a complaint of diffuse, painful redness on both legs (Fig. 1). The patient did not benefit from topical steroid and antibiotic therapy, and a biopsy was taken from the right thigh of the patient, with a clinical preliminary diagnosis of panniculitis. A minimally inflamed skin tissue was observed microscopically. Complete blood count was normal. ESR was 111 mm/h, and LDH was 794 U/L. No atypical cells were observed in the peripheral blood smear. Flow cytometric studies were not diagnostic. A thigh magnetic resonance imaging (MRI) showed changes consistent with fasciitis and myositis. EMG was normal. One week later, a biopsy was taken with a clinical preliminary diagnosis of morphea, panniculitis, eosinophilic fasciitis, scleromyxedema, and cutaneous lymphoma. In this biopsy, subtle inflammatory changes were mistaken for morphea. Steroid and methotrexate were given to the patient, because the microscopy was mistaken as morphea (Fig. 2). Upon the progression of clinical findings, the biopsy was re-examined, and atypical lymphoid cells were noted in the lumens of deep vascular structures at high power magnification. Intravascular large B-cell lymphoma was considered in the case. The biopsy revealed the presence of Pax5, Mum1, CD20 positive, CD31, CD5, CD56, and CD138 negative large lymphoid cells (Figs. 3 A−B, 4 A−B). Large lymphoid cells were observed in the vessel lumens of the striated muscle, and adipose and connective tissue in the form of non-invasive, free tumor embolisms. CD3 was reactive in perivascular lymphoid cells. Staining was observed in 5% of the intravascular large lymphoid cells with CD5. C-myc was positive (> 40%). Bcl-2 was positive, while Bcl-6, CD10, CD30, ALK, EBV, and PDL-1 were negative. Activated (non-germinal center) B-cell type, intravascular large B-cell lymphoma was considered. The bone marrow biopsy was normal. Positron emission tomography (PET), abdominal computed tomography (CT), MRI, and endoscopic examination were normal. R-CHOP (rituximab, cyclophosphamide, doxorubicin, vincristine, prednisolone) treatment was initiated.

## Discussion

Firstly described by Pleger and Tappeiner in 1959 as being of endothelial origin, IVBCL, which accounts for less than 1% of all lymphomas, is characterized by the proliferation of neoplastic cells in the lumen of small vessels.^1^, ^3^, ^4^ The emergence mechanism of this tumor, which prevails in less than one in a million individuals and of which there are approximately 500 cases reported so far, is not known yet.^1^, ^2^, ^5^ The prevalence is slightly higher in males, and the median age is 67−70 years (range, 13−90 years old).^3^, ^4^ It has usually been reported to develop in immunosuppressed patients.^6^

Symptoms differ according to the affected organs and geographical distribution of the patients (Table 1). The central nervous system and cutaneous involvements are the most common.^1^ It was divided into two main types depending on clinical presentations: classical (mostly present in Western countries) and Asian (mostly present in Eastern countries).^1^, ^2^ The classical type is characterized by neurological and dermatological symptoms, while the Eastern type is characterized by hemophagocytic syndrome.^1^, ^2^ In some cases, both types of symptoms are observed, and they are named as the intermediate variant.^1^ Several cases of peripheral organ involvement, including the lungs, kidney, and endocrine organs, and lymph node involvement have been reported.^2^, ^3^

**Table 1:** Major patterns of IVBCL and their symptoms.

Classic (Western)	Asian (Eastern)	Isolated cutaneous
**Cutaneous symptoms**	Hemophagocytic symptoms	**Cutaneous symptoms**
Maculopapular eruption	Bone marrow involvement	Maculopapular eruption
Nodules	Pancytopenia	Nodules
Violaceous plaques	Hepatosplenomegaly	Violaceous plaques
Purpura	Multiorgan failure	Purpura
Ulcers	B symptoms	Ulcers
Orange-peel-like changes		Orange-peel-like changes
Cellulite-like infiltration		Cellulite-like infiltration
**Neurological symptoms**		
Altered mental status		
Sensory or motor deficits		
Generalized weakness,		
Rapidly progressive dementia		
Seizures		
Hemiparesis		
Dysarthria		
Ataxia		
**B symptoms**		
**Peripheral organ involvement**		

Cutaneous manifestations occur in about half of the classical-type IVBCL patients and include maculopapular eruption, nodules, violaceous plaques, purpura, ulcers, orange-peel-like changes, or cellulite-like infiltration.^3^, ^4^ Lesions were most commonly described as nodules, plaques, macules, enduration, and telangiectasia.^7^ Patients who present only with skin involvement are younger age and often female, and their prognosis is better in comparison with classical IVBCL patients.^3^, ^4^ A deep biopsy from the lesion-free skin is recommended, because of the non-superficial involvement.^8^, ^9^ This lymphoma is more common and noticeable in hemangioma-like hypervascular lesions. A biopsy from these hypervascular lesions may be more diagnostic.^8^ Lesions are most commonly observed in the thighs, legs, trunk, arms, and inframammary region.^7^ When a patient has recurrent thrombophlebitis, recurrent erythema nodosum, and erysipelas that do not respond to antibiotic therapy, the possibility of IVBCL should be considered.^7^

The most common laboratory findings are LDH and β2 microglobulin increase.^3^ Prostatic acid phosphatase was investigated as a diagnostic indicator.^3^

The neoplastic lymphoid cells have large vesicular nuclei, narrow cytoplasm, single or multiple prominent nucleoli.^2^, ^5^ One small cell variant has been identified.^3^ It presents a morphological spectrum that ranges from centroblasts to immunoblasts and plasmablasts, including rare forms with anaplastic morphology.^5^ It may appear in cohesive, discohesive, and marginating patterns.^5^ In addition to capillaries and the sinusoids of the liver and bone marrow, the red pulp of the spleen are usually infiltrated with atypical lymphocytes.^4^

These tumors usually express B-cell antigens. CD5 positivity has been reported in 30%−38% of the cases, which have been associated with bone marrow/peripheral blood involvement and thrombocytopenia.^1^ Furthermore, neurological abnormalities have been observed less frequently in CD5-positive cases than in CD10-negative cases.^1^ Only one case with CD30 positivity has been described to date.^9^

Bone marrow biopsy, brain MRI and PET are required for staging.^9^ Some authors suggest taking a random biopsy of normal-looking skin, bone marrow biopsy, and transbronchial lung biopsy as a part of staging.^8^ Patients with IVBCL respond poorly to multi-agent chemotherapy.^3^ The overall response rate of IVBCL patients treated with CHOP is 59% and the three-year survival rate is 33% in Western countries.^5^ The combination of chemotherapy and rituximab has yielded positive results in subsequent studies.^1^ Since Provasi's CD30 positive case relapsed after chemotherapy, it was thought that this marker could be correlated with resistance to chemotherapy in this lymphoma.^9^ In the autopsy case with extensive IVBCL, strong Programmed Death-Ligand 1 (PD-L1) expression was demonstrated on neoplastic lymphocytes.^10^ The above-mentioned finding may have significant therapeutic effects.^10^ The annual survival rate was 22% for other IVBCL cases and 56% in the cutaneous variant.^4^

The diagnosis is more difficult when there is isolated skin involvement. Keeping this entity in mind, performing a careful microscopic examination, and applying new, effective treatment regimens will yield better clinical results in IVBCL cases.

## Financial support

None declared.

## Authors' contributions

Deniz Bayçelebi: Drafting and editing of the manuscript; design and planning of the study; collection, analysis, and interpretation of data; approval of the final version of the manuscript.

Levent Yıldız: Critical review of the manuscript; design and planning of the study; collection, analysis, and interpretation of data; approval of the final version of the manuscript.

Nilgün Şentürk: Intellectual participation in the propaedeutic and/or therapeutic conduct of the studied cases; design and planning of the study; collection, analysis, and interpretation of data; approval of the final version of the manuscript.

## Conflicts of interest

None declared.
